# Reproductive Outcomes of Conventional *In Vitro* Fertilization and Intracytoplasmic Sperm Injection in Patients with Non-Severe Male Infertility Across Poor and Different Sub-Optimal Ovarian Response Categories: A Cohort Study Based on 30,352 Fresh Cycles from 2009–2019

**DOI:** 10.1007/s43032-023-01444-0

**Published:** 2024-01-16

**Authors:** Dina Jiesisibieke, Tian Tian, Xiaxuan Zhu, Shilin Fang, Nan Zhang, Jinxi Ma, Yuqi Xia, Rong Li, Ping Liu, Jie Qiao, Rui Yang

**Affiliations:** 1 Peking University Third Hospital, Beijing, 100191 China; 2 Center for Reproductive Medicine, Department of Obstetrics and Gynecology, Peking University Third Hospital, No. 49 North Huayuan Road, Haidian District, 100191 Beijing China; 3 National Clinical Research Center for Obstetrics and Gynecology, Beijing, 100191 China

**Keywords:** *In vitro* fertilization, Intracytoplasmic sperm injection, Non-severe male infertility, Obstetric outcomes, Low ovarian response

## Abstract

Due to the influence of economic, social and many other factors, there are more and more reproductive problems. Originally introduced for managing male factor infertility, intracytoplasmic sperm injection had become the most commonly used fertilization treatment in the world, with broadened indications including low oocyte yield, prior fertilization failure with conventional *in vitro* fertilization etc. However, academic evidence for better live-birth outcomes of intracytoplasmic sperm injection over conventional *in vitro* fertilization is limited. Thus, we aimed to compare the reproductive outcomes of conventional *in vitro* fertilization and intracytoplasmic sperm injection in patients with non-severe male factor infertility across poor and different sub-optimal ovarian response categories. The fertility rate, implantation rate, clinical pregnancy rate, live birth rate and other obstetric outcomes were mainly compared. Our results showed that independent of the number of oocytes retrieved, intracytoplasmic sperm injection significantly increased the fertilization rate, while conventional *in vitro* fertilization cycles showed a higher implantation rate, clinical pregnancy rate, and live birth rate. No differences were observed in most obstetric outcomes. Our study indicates that poor ovarian response is not an indication for intracytoplasmic sperm injection in couples with non-severe male infertility.

## Backgrounds

Due to the influence of economic, social, and many other factors, the postponement of childbearing age, poor living habits, mental stress, and other factors lead to an increasing number of reproductive problems [1]. According to the 2022 assisted reproductive technology (ART) fact sheet from the European Society of Human Reproduction and Embryology (ESHRE) (Monitoring 2022), one in six couples suffer from infertility problems more than once during their reproductive lifetime across the world. A few years after the first application of conventional *in vitro* fertilization (IVF), the emergence of intracytoplasmic sperm injection (ICSI) has broadened the application of ART [2]. Although ICSI was initially introduced for managing male factor infertility, it has become the most commonly used fertilization treatment worldwide. According to data from the ESHRE in 2018 and the U.S. Centers for Disease Control and Prevention (CDC), ICSI accounted for approximately 39.7% of all cycles in 39 registered European countries and 68.3%–79.3% across all age groups in America [3, 4]. Considering the wider use of ICSI, the American Society of Reproductive Medicine (ASRM) analyzed the outcomes of ICSI for couples with non-male factor infertility in the latest practice committee opinion [5]. It was concluded that academic evidence for better live-birth outcomes of ICSI over conventional IVF is limited [5]. And according to its procedure, ICSI bypasses the natural barriers of the oocyte through directly injecting a single sperm into an oocyte. This invasive operation may increase the risk of the transmission of genetic defects [6, 7]. Collectively, extra time, costs, and possible risks put an additional note of caution in the broader use of ICSI in fertilization treatments [8].

The primary outcome of ART depends on many factors, among which, ovarian response is one of the most important factors [9]. In conventional IVF, fewer retrieved oocytes were related to fertilization failure and a higher cycle cancellation rate [10]. Although the definition of low ovarian response and the criteria for the number of oocytes retrieved after ovarian stimulation have been the subject of debate in ART, the most commonly used criteria were the Bologna criteria by the ESHRE in 2011 and the POSEIDON criteria in recent years [11]. The commonly used definition of poor ovarian response is the retrieval of < 4 oocytes, and suboptimal ovarian response is the retrieval of 4–9 oocytes after ovarian stimulation [11]. Impaired ovarian reserve and poor ovarian response are common infertility factors, in which the reproductive outcome is closely related to the number of oocytes retrieved [12]. Considering this close link, poor ovarian response can indicate ICSI, in theory, to increase the fertilization rate. However, to the best of the authors’ knowledge, there is a paucity of comparisons of the clinical effects of conventional IVF and ICSI across poor (1–3 oocytes retrieved) and suboptimal (4–9 oocytes retrieved) ovarian response categories [13, 14]. There were some limitations in the previous studies, such as limited sample size [15], less detailed study outcomes [16], and failure to conform to standards [17].

Therefore, our study aimed to compare the reproductive outcomes of conventional IVF and ICSI in couples with non-severe male factor infertility across poor (1–3 oocytes retrieved) and different suboptimal (4–6; 7–9 oocytes retrieved) ovarian response categories. Furthermore, we compared the effect of ICSI over conventional IVF on clinical pregnancy rate (CPR), live birth rate (LBR), abortion rate (AR), and preterm delivery rate after adjusting for potential confounding factors, which may provide information for clinical decision-making.

## Materials and Methods

### Subjects and Data

This retrospective study was conducted at the Reproductive Medicine Center of the Peking University Third Hospital, Beijing, China. Based on the inclusion criteria, patients who underwent conventional IVF/ICSI procedures at the Medical Center for Reproductive Medicine of Peking University Third Hospital between 2009 and 2019 were included in this study. Data were collected from the medical records kept by the center. Cycles were included based on fresh embryo transfer cycles. Cycles were excluded from this study based on the following: (1) cycles with missing or incorrect data (n = 6,541; 4.4%); (2) cycles that had > 9 retrieved oocytes or no oocytes (n = 83,316; 55.9%); (3) cycles with severe male infertility including oligospermia, severe oligospermia, azoospermia or other male factors including ejaculatory disorder, male genetic abnormality, necrospermia, or cycles performed TESA, PESA or MESA, or any cycle using donor gametes (n = 26,824; 18.0%); (4) cycles received preimplantation genetic testing (PGT, n = 1,002; 0.7%); (5) cycles performed half-ICSI, rescue ICSI, IVM, OP-IVM and oocyte cryopreservation (n = 1,019; 0.7%). Severe oligospermia and astheno-spermia were defined as: sperm concentration < 10** × **10^6^/ml, and sperm with progressive motility rate (a + b) < 10% in our clinical diagnostic criteria. A total of 30,352 cycles (22,472 conventional IVF cycles and 7,880 ICSI cycles) were included. Based on the number of oocytes retrieved, the included cycles were divided into three groups: 1–3 (n = 8,680), 4–6 (n = 1,071), and 7–9 (n = 11,101).

### Outcome Measures

The primary outcomes included fertilization rate, clinical pregnancy rate (CPR; clinical pregnancy cycles/transplanted cycles), and live birth rate (LBR; live birth cycles/transplanted cycles). Secondary outcomes were embryonic condition (mean number of 2PN embryos, cleavage stage embryos, transferable embryos, and high-quality embryos), implantation rate (IR, the number of gestational sacs/the number of embryos transferred), and abortion rate (AR, abortion cycles/clinical pregnancy cycles). High quality embryos were defined as ≥ 5 G2 on day 3 of culture. Embryos were graded based on their blastomere number, size and symmetry as well as the percentage of fragmentation that was present: G1: cell with even sized blastomeres and without fragmentation; G2: cell with ≤ 10% fragmentation; G3: even sized cell with 10–30% fragmentation; G4: cell with ≥ 30% fragmentation [18].

### Statistical Analysis

All analyses were performed using IBM SPSS Statistics 24. Visual inspection of histograms and normality Q-Q plots were used to check whether all quantitative variables were normally distributed within each type of explanatory variable. The mean values were compared to assess the association between the quantitative outcomes. Independent sample *t*-tests and non-parametric tests were conducted to assess the statistical significance between the study groups. Categorical outcomes between the conventional IVF and ICSI groups were compared using the chi-square test. A binary logistic regression model was used to compare the effect of ICSI over conventional IVF on CPR, LBR, AR, and preterm delivery rate after adjustment for potential confounding factors, which were defined as non-equally distributed between ICSI and conventional IVF in each retrieved oocyte group. Statistical significance was set at P < 0.05.

### Ethical Approval

This study was approved by the Ethics Committee of Peking University Third Hospital for data handling process (No. IRB00006761-M2020007). All the participants and procedures followed the required guidelines and provided informed consent.

## Results

### Characteristics of Patients

**Fig. 1:**
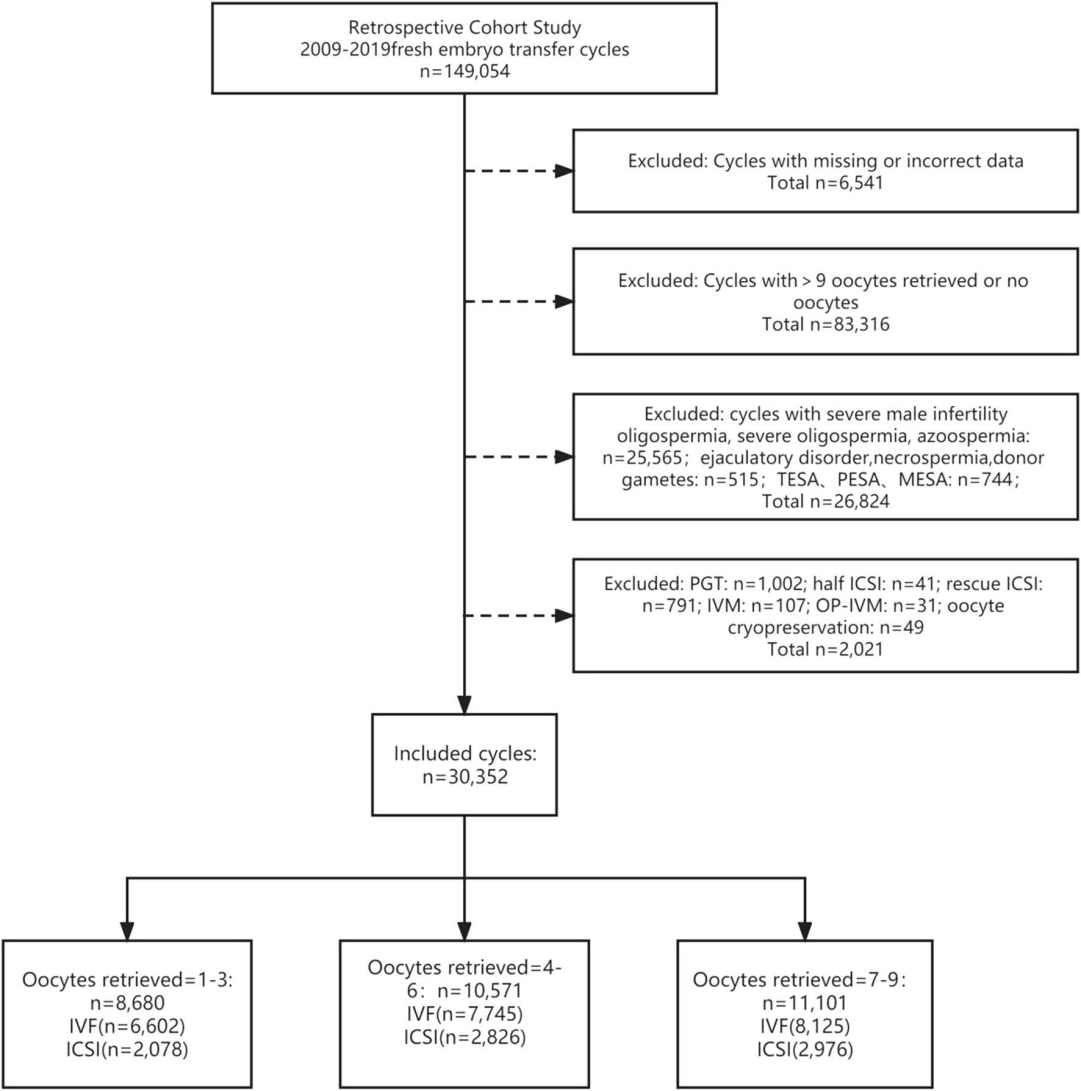
Flowchart of the included population

A total of 149,054 fresh embryo transfer cycles were performed at the Medical Center for Reproductive Medicine of Peking University Third Hospital from to 2009–2019. Finally, we reviewed 30,352 cycles (22,472 conventional IVF cycles and 7,880 ICSI cycles) after screening. The data selection process is illustrated in Fig. 1.

The baseline characteristics of female patients and infertility workup are presented in Table 1. Baseline data were compared between conventional IVF and ICSI cycles in three groups across different ovarian response categories: 1–3, 4–6, and 7–9 oocytes retrieved. In all three groups, patients in the ICSI group were significantly older and had longer durations of infertility than those in the conventional IVF group, whereas body mass index (BMI), AMH, and bFSH levels were similar. ICSI cycles had a significantly higher level of bLH, whereas conventional IVF cycles had higher bE2 levels in groups 1–3 and 4–6 oocytes, respectively. Patients with ≤ 3 oocytes were more likely to suffer from secondary infertility in the conventional IVF group whereas the ICSI group showed the opposite trend in groups with ≥ 4 oocytes. Also, the main infertility factor for patients with ≤ 3 oocytes was diminished ovarian reserve while patients were more likely to suffer from other factors(hyperprolactinemia, unexplained infertility etc.) if ≥ 4 oocytes were retrieved. The ovulation induction protocols varied between conventional IVF and ICSI cycles (p < 0.05).

**Table 1:** Demographic and clinical parameters† of the study population

	**Number of Oocytes Retrieved = 1–3; N = 8680**			**Number of Oocytes Retrieved = 4–6; N = 10,571**			**Number of Oocytes Retrieved = 7–9; N = 11,101**		
	**IVF (N = 6602)**	**ICSI (N = 2078)**	**P**	**IVF (N = 7745)**	**ICSI (N = 2826)**	**P**	**IVF (N = 8125)**	**ICSI (N = 2976)**	**P**
**Age, years**									
Mean (SD)	37.1 ± 5.3	38.0 ± 5.2	** < 0.001**	34.9 ± 5.0	35.5 ± 5.3	**< 0.001**	33.6 ± 4.7	34.1 ± 4.7	**< 0.001**
20–29	560 (8.5)	134(6.4)	**< 0.001**	1089(14.1)	383(13.6)	**< 0.001**	1599(19.7)	479(16.1)	**< 0.001**
30–34	1573(23.8)	412(19.8)		2599(33.6)	855(30.3)		3180(39.1)	1127(37.9)	
35–39	2119(32.1)	631(30.4)		2558(33.0)	876(31.0)		2398(29.5)	969(32.6)	
≥ 40	2350(35.6)	901(43.3)		1499(19.4)	712(25.2)		947(11.7)	401(13.5)	
**BMI, kg/m**^**2**^	23.1 ± 3.7	22.9 ± 3.7	0.094	23.0 ± 3.8	22.8 ± 3.6	0.089	22.8 ± 3.6	22.8 ± 3.6	0.681
**Duration of infertility, years**	5.7 ± 4.8	6.6 ± 5.1	**< 0.001**	5.1 ± 4.1	5.8 ± 4.5	**< 0.001**	4.8 ± 3.8	5.5 ± 4.1	**< 0.001**
**Infertility type, n (%)**									
Primary infertility	2918(44.2)	991(47.7)	0.005	3511(45.3)	1559(55.2)	**< 0.001**	3773(46.4)	1773(59.6)	**< 0.001**
Secondary infertility	3684(55.8)	1087(52.3)		4234(54.7)	1267(44.8)		4352(53.6)	1203(40.4)	
**Infertility factors, n (%)**			.124			** < 0.001**			** < 0.001**
Diminished ovarian reserve	2730(41.4)	884(42.5)		1661(21.4)	645(22.8)		921(11.3)	305(10.2)	
Fallopian tube disorders	966(14.6)	256(12.3)		1698(21.9)	382(13.5)		2173(26.7)	519(17.4)	
Uterine disorders	380(5.8)	124(6.0)		485(6.3)	175(6.2)		483(5.9)	186(6.3)	
Endometriosis	243(3.7)	83(4.0)		357(4.6)	116(4.1)		372(4.6)	112(3.8)	
Others	2283(34.6)	731(35.2)		3544(45.8)	1508(53.4)		4176(51.4)	1854(62.3)	
**AMH(ng/mL)**	0.5(0.2–1.0)	0.5(0.2–1.1)	0.051	1.1(0.6–2.1)	1.1(0.6–2.0)	0.335	1.7(1.1–3.0)	1.8(1.1–2.8)	.621
**bFSH(IU/L)**	8.7(6.5–11.8)	8.6(6.4–12.2)	0.807	7.4(5.7–9.5)	7.5(5.6–9.5)	0.317	6.7(5.3–8.4)	6.8(5.3–8.5)	.886
**bLH(IU/L)**	3.3(2.2–4.7)	3.4(2.0–4.9)	**0.023**	3.2(2.1–4.5)	3.2(2.1–4.6)	0.694			
**bE2(pmol/mL)**	166.0(127.0–217.0)	167.5(120.0–225.0)	0.067	166.0(130.0–214.0)	159.0(126.3–213.8)	**0.003**	163.0(126.0–208.0)	167.0(126.0–217.0)	.233
**Ovarian stimulation treatment methods, n (%)**									
COS	4348(65.9)	1212(58.3)	**< 0.001**	7070(91.3)	2446(86.6)	**< 0.001**	7903(97.3)	2855(95.9)	**< 0.001**
MSP/NC	2254(34.1)	866(41.7)		675(8.7)	380(13.4)		222(2.7)	121(4.1)	

The significant data were presented in bold

*COS* controlled ovarian stimulation, *MSP*mild stimulation protocols, *NC* natural cycle

^†^: Quantitative variables normally distributed were described as mean ± SD. Quantitative variables abnormally distributed were reported as median (interquartile range)

### Comparison of Laboratory Outcomes

Laboratory outcomes in groups with 1–3, 4–6, and 7–9 oocytes retrieved were compared between conventional IVF and ICSI in Table 2. The 2PN fertility rate was higher in ICSI cycles, independent of the number of oocytes retrieved (Group 1:58.1% versus 67.2%; Group 2:60.1% versus 65.1%; Group 3:60.0% versus 66.7%, all P < 0.001). There were more transferable D3 embryos observed in conventional IVF cycles in all three groups (1.5 ± 0.9 versus 1.4 ± 0.8; 3.8 ± 1.4 versus 3.0 ± 1.4; 6.0 ± 1.9 versus 4.7 ± 1.9, all p < 0.001). When ≥ 4 oocytes were retrieved, conventional IVF cycles obtained more 2PN embryos and high-quality embryos (3.0 ± 1.4 versus 2.6 ± 1.4; 4.8 ± 1.9 versus 4.0 ± 1.9; 2.0 ± 1.5 versus 1.7 ± 1.4; 3.2 ± 2.1 versus 2.8 ± 2.0, all p < 0.001) and higher transplantation rate (82.3% versus 79.2%; 88.5% versus 84.8%). Most cases received Day 3 embryos transfer. In groups of ≤ 6 oocytes were retrieved, no differences were observed in the proportion of Day 3/Day 5 embryo transfer between IVF and ICSI groups. When ≥ 7 oocytes were retrieved, ICSI cycles had higher proportion of Day 3 embryos transfer.

**Table 2:** Laboratory outcomes of IVF and ICSI in non-male factor cycles

	**Number of Oocytes Retrieved = 1–3; N = 8680**			**Number of Oocytes Retrieved = 4–6; N = 10,571**			**Number of Oocytes Retrieved = 7–9; N = 11,101**		
	**IVF (N = 6602)**	**ICSI (N = 2078)**	**P**	**IVF (N = 7745)**	**ICSI (N = 2826)**	**P**	**IVF (N = 8125)**	**ICSI (N = 2976)**	**P**
**2PN fertility rate (%)**	58.1(7855/13526)	2541/3783(67.2)	**< 0.001**	60.1(23,472/39061)	65.1(7454/11455)	**< 0.001**	60.0(39,065/65059)	66.7(12,469/18718)	** < 0.001**
**Number of 2PN fertilized embryos**	1.2 ± 0.9	1.2 ± 0.8	0.125	3.0 ± 1.4	2.6 ± 1.4	**< 0.001**	4.8 ± 1.9	4.0 ± 1.9	**< 0.001**
**Number of transferable D3 embryos**	1.5 ± 0.9	1.4 ± 0.8	**< 0.001**	3.8 ± 1.4	3.0 ± 1.4	**< 0.001**	6.0 ± 1.9	4.7 ± 1.9	**< 0.001**
**Number of high quality embryos**	0.8 ± 0.8	0.8 ± 0.8	0.312	2.0 ± 1.5	1.7 ± 1.4	**< 0.001**	3.2 ± 2.1	2.8 ± 2.0	**< 0.001**
**Transplantation rate, n (%)**	3705/6602(56.1)	1158/2078(55.7)	.761	6378/7745(82.3)	2239/2826(79.2)	**< 0.001**	7190/8125(88.5)	2525/2976(84.8)	**< 0.001**
Proportion of Day 3 transfers	3601/3705(97.2)	1138/1158(98.3)	.053	6161/6378(96.6)	2168/2239(96.8)	.677	6908/7190(96.1)	2451/2525(97.1)	.045
Proportion of Day 5 transfers	104/3705(2.8)	20/1158(1.7)		217/6378(3.4)	71/2239(3.2)		282/7190(3.9)	74/2525(2.9)	
**Numbers of embryos transferred**	1.5 ± 0.6	1.5 ± 0.6	.088	2.0 ± 0.5	1.9 ± 0.6	**< 0.001**	2.1 ± 0.5	2.1 ± 0.5	.931

The significant data were presented in bold

2PN fertility rate = (IVF: 2PN/IVF oocyte; ICSI: 2PN/ MII oocyte); Transplantation rate = Transplanted cycles/total cycle;

### Comparison of Clinical Outcomes

The clinical outcomes were also compared between the conventional IVF and ICSI groups (Table 3). Conventional IVF cycles showed a higher implantation rate (IR, 17.6% versus 13.2%; 22.2% versus 20.2%; 26.3% versus 23.9%, p < 0.05) and clinical pregnancy rate (CPR, 23.2% versus 17.2%; 34.3% versus 30.9%; 41.3% versus 38.6%, p < 0.05) than ICSI cycles independent of the number of oocytes retrieved. The live birth rate was significantly higher in the conventional IVF cycles than in the ICSI cycles (LBR, 17.1% versus 12.6%; 26.6% versus 24.2%; 32.7% versus 30.8%, p < 0.05). No differences were observed in most obstetric outcomes, including abortion rate, early abortion rate, twin births rate, cesarean delivery rate, gestational age, and congenital malformations rate, between conventional IVF and ICSI in all three groups (p ≥ 0.05). However, conventional IVF cycles showed higher preterm delivery rates and lower birth weights in groups with 4–6 and 7–9 oocytes retrieved. If twin births were excluded, this result was only significant in the group with 7–9 oocytes retrieved (8.5% versus 5.5%, p = 0.022; 3297.2 ± 540.3 versus 3359.4 ± 523.5, p = 0.016). Interestingly, more female infants born in ICSI cycles with 4–6 oocytes were retrieved (male:53.4% vs. 48.7%; female:46.6%vs. 51.3%, p = 0.038).

**Table 3:** Clinical outcomes of IVF and ICSI in non-male factor cycles

		**Number of Oocytes Retrieved = 1–3; N = 8680**			**Number of Oocytes Retrieved = 4–6; N = 10,571**			**Number of Oocytes Retrieved = 7–9; N = 11,101**		
		**IVF (N = 6602)**	**ICSI (N = 2078)**	**P**	**IVF (N = 7745)**	**ICSI (N = 2826)**	**P**	**IVF (N = 8125)**	**ICSI (N = 2976)**	**P**
**IR, n (%)**		968/5505(17.6)	223/1685(13.2)	**< 0.001**	2767/12437(22.2)	856/4235(20.2)	**0.006**	3897/14834(26.3)	1247/5207(23.9)	**0.001**
**CPR, n (%)**		861/3705(23.2)	199/1158(17.2)	**< 0.001**	2188/6378(34.3)	692/2239(30.9)	**.003**	2968/7190(41.3)	975/2525(38.6)	**.019**
**AR, n (%)**		199/861(23.1)	49/199(24.6)	.650	414/2188(18.9)	117/692(16.9)	.234	507/2968(17.1)	174/975(17.8)	.584
**Early abortion rate, n (%)**		161/861(18.7)	43/199(21.6)	.348	338/2188(15.4)	95/692(13.7)	.270	373/2968(12.6)	125/975(12.8)	.836
**LBR, n (%)**		633/3705(17.1)	146/1158(12.6)	**< 0.001**	1695/6378(26.6)	542/2239(24.2)	**.031**	2348/7190(32.7)	777/2525(30.8)	**.044**
**Twin births rate, n (%)**		70/633(11.0)	17/146(11.6)	0.859	395/1695(23.3)	111/542(20.5)	.188	619/2348(26.4)	182/777(23.4)	.125
**Total Preterm delivery rate, n (%)**		75/633(11.8)	12/146(8.2)	0.258	294/1695(17.3)	63/542(11.6)	**.002**	418/2348(17.8)	106/777(13.6)	**.008**
**Preterm delivery rate of single births**		45/563(8.0)	7/129(5.4)	.372	126/1300(9.7)	29/431 (6.7)	.062	147/1728(8.5)	33/595(5.5)	**.022**
**Preterm delivery rate of twin births**		30/70(42.9)	5/17(29.4)	.412	168/395(42.5)	34/111(30.6)	.028	271/619(43.8)	73/182(40.1)	.395
**Total Cesarean delivery rate, n (%)**		491/633(77.6)	118/146(80.8)	.464	1293/1695(76.3)	400/542(73.8)	.231	1813/2348(77.2)	578/777(74.4)	.155
**Cesarean delivery rate of single births, n (%)**		424/563(75.3)	101/129(78.3)	.556	927/1300(71.3)	294/431(68.2)	.227	1225/1728(70.9)	409/595(68.7)	.439
**Cesarean delivery rate of twin births, n (%)**		67/70(95.7)	17/17(100)	1.000	366/395(92.7)	106/111(95.5)	.392	587/619(94.8)	169/182(92.9)	.395
**Total Gestational age (weeks), mean ± SD**		38.2 ± 1.7	38.3 ± 1.5	.513	37.9 ± 2.2	38.0 ± 2.1	.338	37.9 ± 2.2	38.0 ± 2.1	.106
**Gestational age of single births (weeks), mean ± SD**		38.4 ± 1.5	38.6 ± 1.2	.262	38.4 ± 2.0	38.5 ± 1.7	.315	38.5 ± 1.9	38.6 ± 1.6	.060
**Gestational age of twin births (weeks), mean ± SD**		36.1 ± 2.0	36.6 ± 3.0	.380	36.2 ± 2.2	36.5 ± 2.0	.283	36.1 ± 2.1	36.3 ± 2.1	.280
**Total Newborn weight(kg), mean ± SD**		3163.8 ± 575.3	3187.3 ± 607.4	.646	3008.3 ± 664.3	3063.9 ± 609.3	.048	2967.9 ± 652.1	3037.5 ± 660.7	**.005**
**Newborn weight of single births(kg), mean ± SD**		3309.9 ± 500.2	3366.3 ± 468.9	.246	3292.5 ± 568.4	3294.5 ± 505.3	.947	3297.2 ± 540.3	3359.4 ± 523.5	**.016**
**Newborn weight of twin births (kg), mean ± SD**		2676.1 ± 472.5	2623.7 ± 550.7	.699	2629.1 ± 520.1	2739.1 ± 527.0	.052	2600.5 ± 495.0	2619.6 ± 472.4	.645
**Live birth Gender**	**Male**	387/690(56.1)	76/160(47.5)	.053	1099/2059(53.4)	316/649(48.7)	**.038**	1566/2936(53.3)	472/947(49.8)	.061
	**Female**	303/690(43.9)	84/160(52.5)		960/2059(46.6)	333/649(51.3)		1370/2936(46.7)	475/947(50.2)	
**Congenital malformations rate, n (%)**		3/633(0.5)	0/146(0.0)	0.403	5/1695(0.2)	2/542(0.4)	0.679	5/2348(0.2)	3/777(0.4)	.418

The significant data were presented in bold

^‡^Multiple births excluded; §: Because of a loss to follow-up (about 1%) in a few patients for obstetric outcomes, the number of cycles used to calculate LBR is less than the number of cycles originally included. *IR* implantation rate, *CPR* clinical pregnancy rate, *AR* abortion rate, *LBR* live birth rate

2PN fertility rate = (IVF: 2PN/IVF oocyte; ICSI: 2PN/ MII oocyte); Transplantation rate = Transplanted cycles/total cycle; IR = number of gestational sac/number of transplanted embryos; CPR = clinical pregnancy cycles/transplanted cycles; AR = abortion cycles/clinical pregnancy cycles; Early abortion rate = cycles ≤ 12 weeks of pregnancy/clinical pregnancy cycles; Congenital malformations rate = Abortion malformation + fetal malformation cycles/live birth cycles); Multiple fetus rate = cycles ≥ 2 fetus/clinical pregnancy cycles; LBR = live birth cycles/transplanted cycles; Multiple births rate = cycles ≥ 2 newborn/live birth cycles; Preterm delivery rate = preterm delivery cycles/live birth cycles; Cesarean delivery rate = cesarean delivery cycles/live birth cycles;

### Binary Logistic Regression for Effect Comparison

We compared the effect of ICSI over conventional IVF on CPR, LBR, AR, and preterm delivery rate after adjusting for potential confounding factors (Table 4). The results demonstrated that only in cycles with 1–3 oocytes retrieved, ICSI was associated with a significant decrease in CPR (OR = 0.763, 95% CI, 0.636, 0.916, p = 0.004) and LBR (OR = 0.795, 95% CI, 0.646, 0.979, p = 0.030). There was no evidence that fertilization methods were associated with AR (OR = 1.033, 95% CI, 0.709, 1.505; OR = 0.869, 95% CI, 0.684, 1.104; OR = 1.048, 95% CI, 0.859, 1.278, all p > 0.05). The results also indicated that ICSI was associated with a significant decrease in the preterm delivery rate, independent of the inclusion of multiple births when ≥ 4 oocytes were retrieved.

**Table 4:** The binary logistic regression to compare the effect of ICSI with IVF on CPR, LBR, AR and preterm delivery rate

Group	Number of Oocytes Retrieved = 1–3; N = 8680		Number of Oocytes Retrieved = 4–6; N = 10,571		Number of Oocytes Retrieved = 7–9; N = 11,101	
Variables	OR (95%CI)	Value	OR (95%CI)	P-Value	OR (95%CI)	P-Value
CPR^†^	0.744(0.621–0.892)	**0.001**	0.916(0.821–1.023)	0.120	1.918(0.833–1.012)	0.087
LBR^‡^	0.772(0.628–0.949)	**0.014**	0.940(0.835–1.059)	0.311	0.939 (0.847–1.041)	0.233
AR‡	1.051 (0.721–1.533)	0.795	0.865 (0.680–1.099)	0.235	1.048 (0.859–1.278)	0.646
preterm delivery rate^§^	0.580 (0.290–1.163)	0.125	0.659 (0.481–0.904)	**0.010**	0.768 (0.591–0.997)	0.047
preterm delivery rate*	0.702(0.365–1.349)	0.288	0.618(0.458–0.833)	**0.002**	0.762(0.601–0.967)	**0.026**

The significant data were presented in bold

Adjusted potential confounding factors:

^†^: age, infertility type (Primary infertility; Secondary infertility), infertility years, body mass index, ovarian stimulation treatment methods (COS/MSP; NC), infertility factors (Diminished ovarian reserve; Fallopiantube disorders; Uterine disorders; Endometriosis; Others), number of embryos transferred, embryo transferring day

^‡^: age, infertility type (Primary infertility; Secondary infertility), infertility years, body mass index, ovarian stimulation treatment methods (COS/MSP; NC), infertility factors (Diminished ovarian reserve; Fallopiantube disorders; Uterine disorders; Endometriosis; Others), number of embryos transferred, embryo transferring day

^§^: age, infertility type (Primary infertility; Secondary infertility), infertility years, body mass index, ovarian stimulation treatment methods (COS/MSP; NC), infertility factors (Diminished ovarian reserve; Fallopiantube disorders; Uterine disorders; Endometriosis; Others), number of embryos transferred, embryo transferring day, Multiple births

^*^: age, infertility type (Primary infertility; Secondary infertility), infertility years, body mass index, ovarian stimulation treatment methods (COS/MSP; NC), infertility factors (Diminished ovarian reserve; Fallopiantube disorders; Uterine disorders; Endometriosis; Others), number of embryos transferred, embryo transferring day

## Discussion

The results of our study demonstrated that while ICSI cycles reached higher fertilization rates, conventional IVF cycles still resulted in higher clinical pregnancy rates and live birth rates in patients with non-severe male factor infertility across poor and different suboptimal ovarian response categories, which were more significant when ≤ 3 oocytes were retrieved. No significant differences were found in most obstetric outcomes across the different ovarian response categories.

The number of ART cycles performed has grown by 5–10% each year in many developed countries over the last few years. ICSI plays a significant role in broadening the application of ART by treating male factor infertility. It is a micromanipulation technique that helps the abnormal spermatozoon bypass natural barriers around the oocyte by inserting the spermatozoon into the cytoplasm [19]. ICSI has been considered to decrease the occurrence of fertilization failure [5]. A retrospective study based on 62,641 stimulated fresh cycles of the POR cohort suggested that the fertilization rate was lower in conventional IVF cycles [16]. However, another retrospective study revealed that in patients with ≤ 4 oocytes retrieved, the fertilization rate was better via conventional IVF, whereas implantation rates, live birth rates, and miscarriage rates were similar [15]. Despite its debatable efficacy and safety, ICSI has become the most commonly used ART technique worldwide, with broadened indications including low oocyte yield, prior fertilization failure with conventional IVF, and advanced maternal age [20]. ICSI has undergone relatively strict indications in China and is not as commonly used in European countries and America [21]. Low oocyte yield is a suitable case because fewer oocytes retrieved are related to fertilization failure [10, 22]. Our results showed that ICSI significantly increased the fertilization rate among poor and suboptimal ovarian response cycles, which is in line with most previous studies.

Several studies have compared conventional IVF and ICSI in terms of embryonic outcomes [23–25]. In most studies, there was no significant difference in the proportion of good-quality embryos and 2PN embryos [24, 25]. However, in another study, conventional IVF cycles showed a higher cleavage rate, which is consistent with our results. Our results demonstrated that more transferable D3 embryos were observed in conventional IVF cycles in all the groups. When ≥ 4 oocytes were retrieved, the conventional IVF cycles resulted in more 2PN and high-quality embryos. This may help explain the better reproductive outcomes in conventional IVF cycles, as the internal developmental potential of oocytes is a critical factor in the process of embryonic development.

In terms of post-fertilization reproductive outcomes, our result was consistent with some previous studies, that is conventional IVF showed significant advantages over ICSI in terms of CPR and LBR [16, 26, 27]. Possible reasons why ICSI improved the fertilization rate but was inferior to conventional IVF in IR, CPR, and LBR have been discussed [28–32]. Unpredictable oocyte damage brought by invasiveness of ICSI has always been a key point. First, the injection needle may damage the cytoplasm of oocytes and cause clusters of smooth endoplasmic reticulum or vacuoles, which may influence the oocyte survival [28, 29]. Furthermore, the inaccurate positioning of the injection needle may do damage to the metaphase II (MII) spindle, leading to an increased risk of producing aneuploid embryos [30]. The reproductive outcome is closely related to the number and quality of oocytes retrieved, and oocytes quality is more likely to be impaired by ICSI procedure in patients with poor ovarian response. Apart from the better clinical outcomes, conventional IVF also shows advantages in lower costs and less time consuming [33]. Thus, the choice of the treatment should be considered comprehensively, both in the efficacy and efficiency side, in the absence of the male factor.

Regarding the majority of obstetric outcomes, another study based on subgroup analysis found no significant differences in multiple birth rate, cesarean delivery rate, and gestational age [34]. Significant decreases were noted in the preterm delivery rate and low birth weight in infants born after ICSI treatment [35, 36]. Similar to these studies, our results demonstrated that multiple fetal, abortion, early abortion, congenital malformations, and cesarean delivery rates, and gestational weeks were similar in each subgroup, but lower preterm delivery rates and higher birth weights in ICSI cycles were observed when ≥ 4 oocytes were retrieved. Wennerholm et al. [37] also concluded that ICSI had a lower incidence of preterm delivery and low birth weight, suggesting that ICSI had a better obstetric outcome. A possible reason for this is that ICSI is usually related to poor semen quality in men, while the reproductive situation in women is better. Therefore, the difference is more significant especially in women with a better ovarian response (≥ 4 oocytes retrieved).

Another finding is the impact of the fertilization method on the gender balance of infants born after ART. According to our results, more male newborns were born after conventional IVF cycles than after ICSI cycles, and the opposite was true for female infants. A similar study from the UK demonstrated that more female infants were born after ICSI, while conventional IVF increased the number of male births, which is consistent with our study [38]. Several hypotheses regarding the underlying mechanism have been proposed in previous studies, including bias towards females when performing sperm selection in ICSI cycles and reduced normally functioning sperm with the Y chromosome caused by the invasive operation during ICSI [39, 40]. Given the widespread use of ART, its long-term effect on sex imbalance should be a concern; thus, further research is needed.

Our study had several strengths and limitations. First, it was based on a large cohort of 30,352 fresh cycles from 2009–2019. Furthermore, our study focused on couples with non-severe male infertility and poor ovarian response, for whom the indication of ICSI has always been the subject of debate. The information used in this study was sourced from hospital records to ensure the detail and accuracy of the data. However, there are some limitations. For example, cycles and not patients have been analyzed due to the condition of hospital records. And our study was limited by the lack of randomization due to the research type; hence, prospective randomized controlled trials are required to provide a higher level of evidence.

## Conclusion

In conclusion, in the presence of a poor ovarian response, our results showed that even if ICSI increased the fertilization rate, conventional IVF exhibited significant advantages over ICSI in IR, CPR, and LBR for patients with non-severe male infertility. Furthermore, the advantages of conventional IVF are independent of the number of eggs retrieved. There were no significant differences in most of the obstetric outcomes. Therefore, considering that the goal of treatment is live birth, poor ovarian response is not an indication of ICSI in couples with non-severe male infertility.

## Data Availability

The datasets generated during and/or analyzed during the current study are available from the corresponding author on reasonable request.

## Abbreviations

ICSI: Intracytoplasmic sperm injection
conventional IVF: Conventional *in vitro* fertilization
ART: Assisted Reproductive Technology
IR: Implantation rate
CPR: Clinical pregnancy rate
LBR: Live birth rate
AR: Abortion rate
